# Intermittent Radioligand Therapy with ^177^Lu-PSMA-617 for Oligometastatic Castration-Resistant Prostate Cancer

**DOI:** 10.3390/cancers15184605

**Published:** 2023-09-17

**Authors:** Nicolai Mader, Christina Schoeler, Niloufar Pezeshkpour, Konrad Klimek, Daniel Groener, Christian Happel, Nikolaos Tselis, Philipp Mandel, Frank Grünwald, Amir Sabet

**Affiliations:** 1 Department of Nuclear Medicine, University Hospital Frankfurt, Theodor-Stern-Kai 7, 60590 Frankfurt am Main, Germany; nicolai.mader@kgu.de (N.M.); christina.nguyenngoc@kgu.de (C.S.); n.pezeshkpour@yahoo.com (N.P.); konrad.klimek@kgu.de (K.K.); daniel.groener@kgu.de (D.G.); christian.happel@kgu.de (C.H.); frank.gruenwald@kgu.de (F.G.); 2 Department of Radiation Oncology, University Hospital Frankfurt, Theodor-Stern-Kai 7, 60590 Frankfurt am Main, Germany; nikolaos.tselis@kgu.de; 3 Department of Urology, University Hospital Frankfurt, Theodor-Stern-Kai 7, 60590 Frankfurt am Main, Germany; philipp.mandel@kgu.de

**Keywords:** prostate-specific membrane antigen (PSMA), ^177^Lu-PSMA-617, radioligand therapy (RLT), intermittent treatment, metastatic castration-resistant prostate cancer (mCRPC)

## Abstract

**Simple Summary:**

Radioligand therapy (RLT) usually consists of predefined cycles of ^177^Lu-PSMA-617 every 6–8 weeks. Although side-effects of RLT are considered well tolerable, cumulative absorbed doses in kidneys and an increased probability of chronic renal failure in responders remain concerns. The aim of our retrospective study, therefore, was to assess the efficacy and safety of intermittent, response-adapted RLT with ^177^Lu-PSMA-617 in oligometastatic mCRPC patients. Intermittent RLT with ^177^Lu-PSMA-617 is effective and can result in long-lasting disease control in selected patients even when including multiple treatment-free periods.

**Abstract:**

^177^Lu-PSMA-617 radioligand therapy (^177^Lu-PSMA-RLT) in patients with metastatic castration-resistant prostate cancer (mCRPC) currently consists of 4–6 cycles of 6.0–7.4 GBq of ^177^Lu-PSMA-617 each every 6–8 weeks. While safety and efficacy could be demonstrated in larger prospective trials irrespective of the tumor burden at ^177^Lu-PSMA RLT initiation, increased renal absorbed doses due to a reduced tumor sink effect in early responding, oligometastatic mCRPC patients pose difficulties. Response-adapted, dose distributing, intermittent treatment with up to six cycles has not been routinely performed, due to concerns about the potential loss of disease control. Treatment was discontinued in 19 early-responding patients with oligometastatic tumor burden after two (IQR 2–3) cycles of ^177^Lu-PSMA-RLT and 6.5 ± 0.7 GBq per cycle and resumed upon ^68^Ga-PSMA-11-PET/CT-based progression (according to the PCWG3 criteria). Subsequent treatment breaks were imposed if a PSMA-based imaging response could be achieved. A total of five (IQR 3–6) cycles reaching a cumulative activity of 32 ± 11 GBq were applied. A routine blood work-up including blood counts and liver and renal function was measured throughout the ^177^Lu-PSMA-RLT and follow-up to grade toxicity according to CTCAE v5.0 criteria. Survival outcome was calculated based on the Kaplan–Meier method. In total, treatment-free periods of 9 (IQR 6–17) cumulative months and the application of ^177^Lu-PSMA-RLT cycles over 16 (IQR 9–22) months could be achieved. Fifteen (84%) patients responded to subsequent cycles after the first treatment break and in 7/19 (37%) patients, intermittent ^177^Lu-PSMA-RLT consisted of ≥2 treatment breaks. The median PFS was 27 months (95% CI: 23–31) and overall survival was 45 months (95% CI: 28–62). No grade ≥3 hematological or renal toxicities could be observed during the 45 ± 21 months of follow-up. The cumulative mean renal absorbed dose was 16.7 ± 8.3 Gy and 0.53 ± 0.21 Gy/GBq. Intermittent radioligand therapy with ^177^Lu-PSMA-617 is feasible in early-responding patients with oligometastatic disease. A late onset of progression after subsequent cycles and the absence of significant toxicity warrants further investigation of the concept of intermittent treatment in selected patients.

## 1. Introduction

Prostate cancer is one of the most common malignancies in men worldwide [1,2]. Surgery and radiotherapy are the two potentially curative therapeutic options for localized disease. Several patients, however, will progress to metastatic disease and may eventually develop castration-resistance under systemic antiandrogen treatment [3]. Patients with advanced metastatic castration-resistant prostate cancer (mCRPC) have a limited survival despite various treatment options introduced during the last decade [4]. Radioligand therapy with ^177^Lu-labeled prostate-specific membrane antigen (PSMA) ligands (^177^Lu-PSMA-RLT) has emerged as a promising treatment option for mCRPC patients. ^177^Lu-PSMA-RLT allows the selective internal radiation of tumor cells expressing PSMA, whilst sparing the surrounding normal tissue. The currently applied standard treatment regimen consists of 4–6 cycles of 7.4 GBq of ^177^Lu-PSMA-617 per cycle every 6–8 weeks, which proved effective with low overall toxicity [5]. In patients with progressive disease, after completing standard treatment, no increased resistance to ^177^Lu-PSMA has been reported in Re-challenge settings [6,7,8,9].

Adverse events of ^177^Lu-PSMA-RLT are mainly due to a physiological off-target accumulation of radioligands in organs expressing PSMA, especially in kidneys and salivary glands. A negative correlation between physiological PSMA-uptake in critical organs and PSMA-expressing tumor burden has been observed in previous studies [10,11,12,13]. As a result, patients with oligometastatic disease and low tumor burden show an increased absorbed dose per administered activity (GBq/Gy) in critical organs [14]. Moreover, a good prognosis of patients with oligometastatic mCRPC undergoing ^177^Lu-PSMA-RLT permits the manifestation of radiation-induced nephrotoxicity months after the treatment. Therefore, EANM procedure guidelines recommend the distribution of the administered activity over the longest clinically reasonable period [15].

This retrospective study assesses the feasibility of an intermittent approach in patients with good prognosis and an initial excellent response to the first 1–3 cycles of ^177^Lu-PSMA-RLT. Treatment was resumed upon progression on PSMA-based imaging and intermittent ^177^Lu-PSMA-RLT with subsequent treatment breaks continued until completion of the intended six cycles. The impact of intermittent RLT on absorbed dose per administered activity over time as well as on renal function was investigated. The survival outcome of patients was analyzed. 

## 2. Materials and Methods

### 2.1. Patient Characteristics

The retrospective analysis included 19 patients with oligometastatic mCRPC with no visceral involvement, clinical symptoms (ECOG ≤ 2), or previous skeletal events (e.g., bone pain and pathological fractures) at the commencement of ^177^Lu-PSMA-RLT. All patients showed an early PSA response (≥50%) accompanied by a partial response on ^68^Ga-PSMA-11 PET/CT imaging to the first 2 (IQR 2–3) cycles of ^177^Lu-PSMA-RLT, which resulted in the first treatment break. Intermittent ^177^Lu-PSMA-RLT was resumed upon PSA progression confirmed by progressive disease on ^68^Ga-PSMA-11 PET/CT imaging. The treatment scheme was reiterated until completion of the intended six cycles. Treatment was performed on a compassionate use basis under the German Pharmaceutical Act §13 (2b) and the initiation of treatment was decided by a multidisciplinary tumor board of experts. All patients had progressive, PSMA-expressing disease on ^68^Ga-PSMA-11 PET/CT imaging before initiating ^177^Lu-PSMA-RLT. Other prerequisites for ^177^Lu-PSMA-RLT were an estimated glomerular filtration rate (eGFR) of >30 mL/min/1.73 m^2^, white blood cells ≥ 2.00 × 10^9^/L, platelets ≥ 75 × 10^9^/L, and hemoglobin (Hb) ≥ 8.0 g/dL. At baseline, 15/19 (79%) patients showed a mild bone marrow impairment of CTCAE grade 1. Eleven patients presented with anemia, one patient with leukopenia, and three patients with anemia and thrombocytopenia. A reduced GFR of grade 1 at baseline was observed in one patient. Baseline characteristics are outlined in Table 1.

A written consent after being thoroughly informed about the risks and side-effects of the therapy and a consent to the publication of their data in accordance with the declaration of Helsinki were obtained from all patients. This study was approved by the local Institutional Review Board (ethics committee permission number 310/18).

### 2.2. Radioligand Therapy with ^177^Lutetium-PSMA-617

ABX (Advanced Biochemical Compounds GmbH, Radeberg, Germany) provided the PSMA-617 ligand, which was labeled in-house with ^177^LuCl^3^ (ITM Isotopen Technologien München AG, Garching/Munich, Germany). Salivary glands were cooled with ice packages for 2 h beginning 30 min before the administration to reduce blood flow and decrease ^177^Lu-PSMA-617 accumulation in salivary glands. An infusion of 1000 mL of 0.9% NaCl solution preceded and followed the administration (30–60 s intravenous injection) of ^177^Lu-PSMA-617. Renal dosimetry was conducted using planar whole-body scintigraphy, computed tomography for renal mass determination, and single-photon emission computerized tomography (SPECT) performed at 24 h, 48 h, and 72 h p.i. during the in-patient procedure at the nuclear medicine therapy ward [16,17]. 

### 2.3. Biochemical and Imaging Analysis

Biochemical response was categorized as response (≥50% decline 12 weeks after treatment initiation), progression (≥25% increase exceeding 2 ng/mL, confirmed through a second measurement ≥3 weeks apart, according to the PCWG3 criteria [18]), and stable (values between <50% decline and <25% increase). PSMA-based imaging response was assessed according to the consensus on ^68^Ga-PSMA-11 PET/CT imaging response [19]: partial response (PR, reduction in uptake and tumor PET volume by >30%), stable disease (SD, uptake and tumor PET volume ± ≤ 30%; no new lesions), and progression (PD, appearance of >2 new lesions or uptake or tumor PET volume ≥30% increased). 

Imaging-based progression-free survival (PFS) was defined as the time interval between initiation of ^177^Lu-PSMA-RLT and progression on ^68^Ga-PSMA-11 PET/CT with no PSMA-based imaging response (PR or SD) to subsequent treatment cycles. The time from treatment initiation to death from any cause was defined as overall survival (OS); censoring was performed if the patient was alive at the time of analysis. PFS and OS were determined using the Kaplan–Meier method (log-rank testing).

### 2.4. Safety Assessment

Hematological (hemoglobin (Hb), white blood cells (WBC), and platelets (PLT)) and renal (eGFR) toxicity evaluation was performed at baseline, prior to each therapy cycle, 2–4 weeks after each cycle, and in 4–12 week intervals throughout treatment-free periods and follow-up. The severity of adverse events was graded based on Common Terminology Criteria for Adverse Events (CTCAE), version 5.0, with grade ≥ 3 toxicities considered significant. A modified, patient-self-reported eight-item xerostomia questionnaire was used to evaluate mouth dryness at every ^177^Lu-PSMA-RLT cycle [20]. The ECOG scale was used to assess the patients’ performance status.

### 2.5. Statistical Analysis

Statistical analyses were performed using SPSS 28.0 (IBM, Armonk, NY, USA). GraphPad Prism version 10.0 (GraphPad Software, San Diego, CA, USA) was used to plot graphs. The significance level was set two-sided at *p* < 0.05. The results are presented as a median with interquartile range (IQR) or mean ± standard deviation for continuous variables, and categorical variables are presented as frequencies with respective percentages. The paired-samples *t*-test was used to compare intraindividual changes in continuous biochemical parameters, and independent-samples t-test was used to compare changes in continuous biochemical parameters in two groups. 

## 3. Results

**Figure 1:**
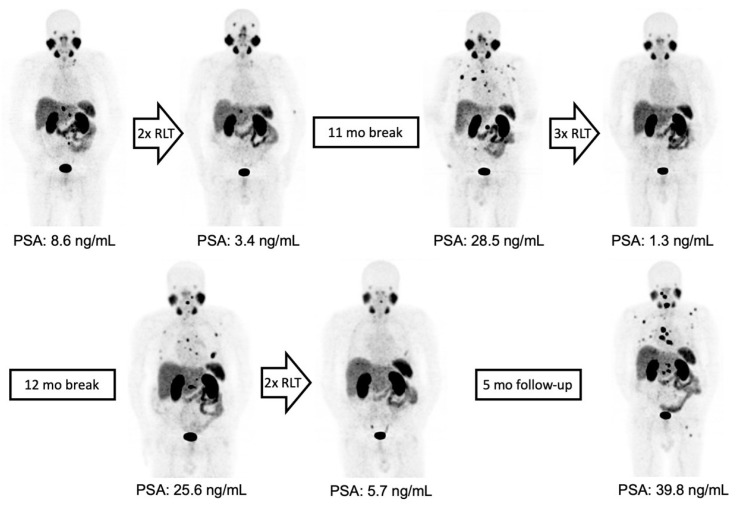
From left to right: maximum-intensity projections (MIPs) of ^68^Ga-PSMA-11 PET/CT images of a patient with initially oligofocal lymph node metastasis and an early partial response with very low residual disease after two treatment cycles. Intermittent ^177^Lu-PSMA-RLT was resumed after 11 months, yielding a repeated response with a second treatment-free interval of 12 months. ^177^Lu-PSMA-RLT duration from the first to last cycle was 33 months and PFS was 42 months.

**Figure 2:**
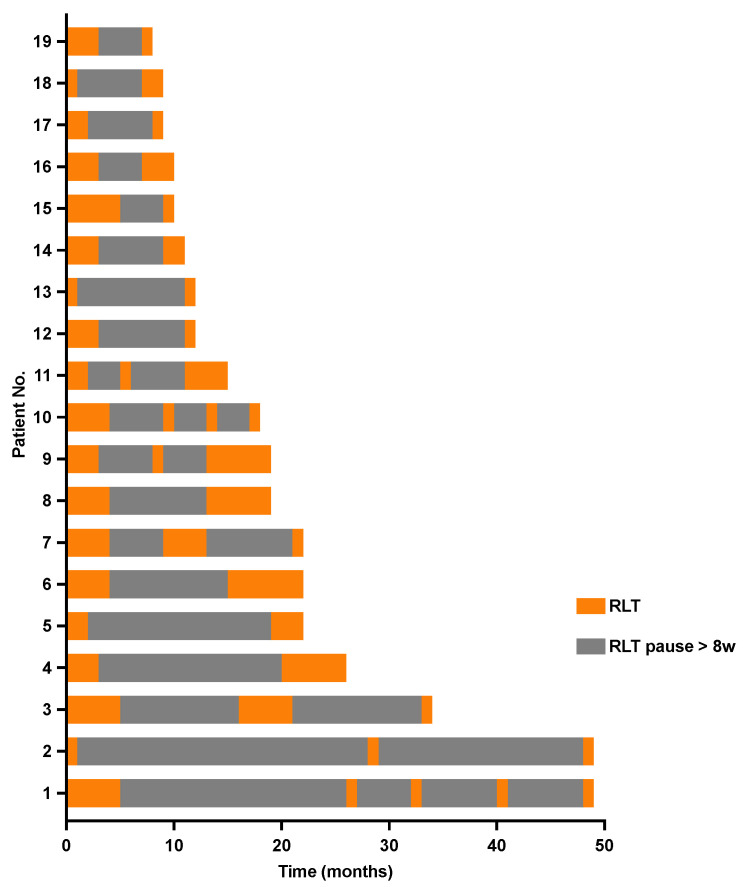
Swimmer plot depicting individual treatment courses after initiation of intermittent radioligand therapy with ^177^Lutetium-PSMA-617 and the respective ^177^Lu-PSMA RLT-free periods.

Nineteen mCRPC patients underwent intermittent ^177^Lu-PSMA-RLT with a total of five (IQR 3–6) cycles of 6.5 ± 0.7 GBq of ^177^Lu-PSMA-617 per cycle and a cumulative activity of 32 ± 11 GBq. The first treatment break occurred after a median of 2 (IQR 2–3) initial cycles, followed by a median of 3 (IQR 2–4) cycles during further intermittent ^177^Lu-PSMA-RLT. Seven (37%) patients could benefit from ≥2 treatment breaks (Figure 1). A mean of 9 (IQR 6–17) treatment-free months during a total treatment period of 16 (IQR 9–22) months could be achieved. The intermittent ^177^Lu-PSMA-RLT course of each patient after a mean follow-up period of 45 ± 21 months is displayed in Figure 2.

### 3.1. Response to Intermittent ^177^Lu-PSMA-RLT

**Figure 3:**
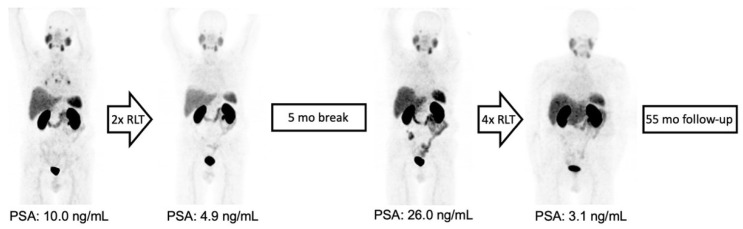
From left to right: MIP of ^68^Ga-PSMA-11 PET/CT images of a patient with initially oligofocal lymph node and bone metastasis and an early partial response with very low residual disease after two treatment cycles. Intermittent ^177^Lu-PSMA-RLT was resumed after 5 months, leading to a repeated response upon completion of six cycles with a total treatment duration of 17 months and OS of 55 months due to an unrelated cause of death.

**Figure 4:**
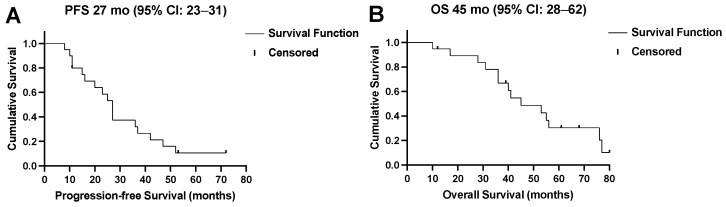
(**A**) Progression-free survival (PFS) and (**B**) overall survival (OS) of all patients in months.

Four (21%) patients showed no response (PD) to the ^177^Lu-PSMA-RLT cycles after the first treatment break, and in two patients (11%), progression after the initial disease stabilization (SD) under subsequent cycles led to treatment discontinuation. Two patients (11%) completed the intended six cycles with a stable disease. In the remaining 11/19 (84%) patients, resuming ^177^Lu-PSMA-RLT resulted in a repeated PR (Figure 3). Altogether, 14/19 (74%) patients died by the time of this analysis. The median PFS was 27 (95% CI: 23–31) months and the OS was 45 (95% CI: 28–62) months. The Kaplan–Meier survival curves are displayed in Figure 4.

### 3.2. Safety

**Figure 5:**
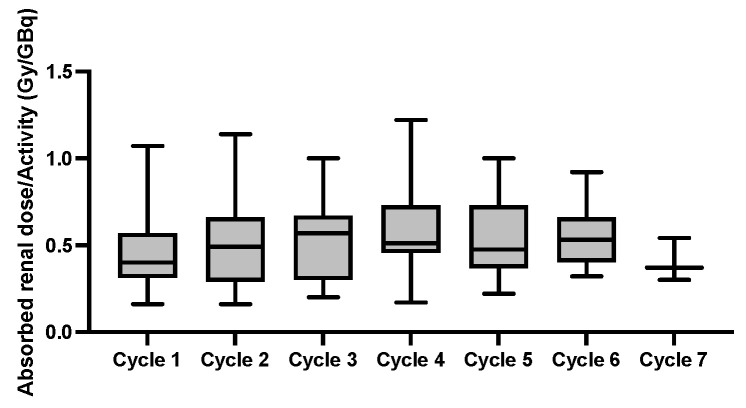
Box plots of the mean absorbed renal dose per activity (Gy/GBq) at each cycle.

No grade ≥ 3 nephrotoxicity was observed but mean eGFR-levels declined from 80.6 ± 18.0 mL/min/1.73 m^2^ at baseline to 73.4 ± 15.6 mL/min/1.73 m^2^ (*p* = 0.055) at the last follow-up. The mean absorbed renal dose was 0.53 ± 0.21 Gy/GBq, with significant absorbed doses from the first cycle onward, resulting in a cumulative absorbed renal dose of 16.7 ± 8.3 Gy (Figure 5). 

A significant correlation, however, between the cumulative renal absorbed dose and administered activity (r_s_ = 0.576, *p* = 0.010) could be observed. Concordantly, in 3/19 (16%) patients with an absorbed renal dose >28 Gy, the administered activity was significantly higher than in patients with <28 Gy of absorbed renal dose (44 ± 7 vs. 30 ± 10 GBq, *p* = 0.036). 

**Figure 6:**
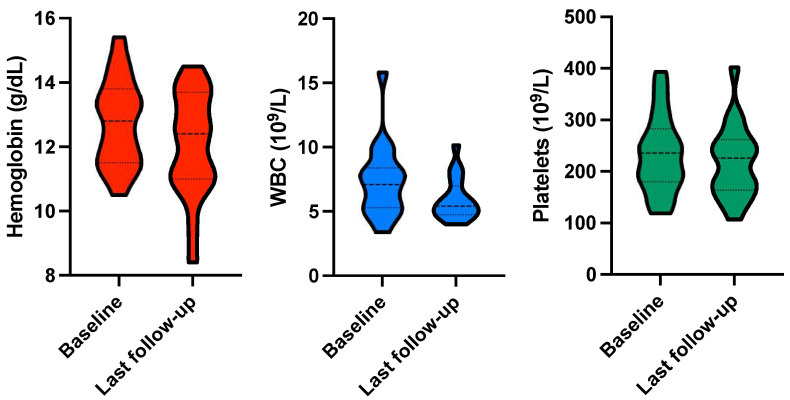
Violin plots for hemoglobin, white blood cells (WBC), and platelets at baseline and upon last follow-up.

No grade ≥ 3 hematological toxicity was observed during intermittent ^177^Lu-PSMA-RLT or follow-up. Nevertheless, Hb, WBC, and PLT showed an absolute decline throughout the course of intermittent ^177^Lu-PSMA-RLT. Mean Hb declined from 12.7 ± 1.4 to 12.0 ± 1.6 g/dL (*p* = 0.021), WBC declined from 7.4 ± 2.8 × 10^9^/L to 5.9 ± 1.6 × 10^9^/L (*p* = 0.005), and PLT declined from 233 ± 76 × 10^9^/L to 218 ± 71 × 10^9^/L (*p* = 0.047; Figure 6).

Significant xerostomia was not detected during ^177^Lu-PSMA-RLT and the follow-up period; one patient developed mild to moderate xerostomia after three treatment cycles, which was not reversible during follow-up. A summary of the adverse events is shown in Table 2.

## 4. Discussion

Applying an intermittent treatment concept with up to four treatment breaks in patients with good prognosis and early response to ^177^Lu-PSMA-RLT, the administered dose of five (IQR 3–6) cycles could be distributed over a treatment period of 16 (IQR 9–22) months. The intermittent approach to ^177^Lu-PSMA-RLT has so far not been generally applied, due to concerns of resistance development to treatment. Previously, in the phase III PRINCE trial, intermittent administration of docetaxel chemotherapy with the intention to reduce toxicity in mCRPC patients showed a non-inferiority of survival and outcome compared to continuous administration [21]. The concept of intermittent therapy, moreover, was evaluated in earlier stages of metastasized prostate cancer. Intermittent androgen deprivation therapy aiming to improve quality of life can be applied, although the treatment outcome remains controversial [22,23]. The response rate in our patient group remained high and 84% of patients responded to subsequent cycles of ^177^Lu-PSMA-RLT after the first treatment-free interval and following progression. Previously described resistance mechanisms do not seem to be associated with an increased number of treatment breaks or distribution of treatment cycles over longer periods [24].

To date, in the ^177^Lu-PSMA-RLT setting, the phase II LuPSMA trial conducted by Hofman et al. included two patients with an exceptional early response, resulting in a treatment break after two cycles, who received the intended additional two cycles upon progression [25]. In a retrospective study, Emmett et al. evaluated the outcome of patients undergoing ^177^Lu-PSMA-RLT with response-guided adjustments to treatment intervals incorporating one treatment break [26]. Patients with a PSA-response >50% after the initial two cycles received a median of 6.1 months (IQR: 3.4–8.7) of ‘treatment holiday’ prior to the subsequent PSA-progression. This subgroup of patients with ‘excellent response’ (*n* = 41) received a total median of three (IQR 2–4) cycles of ^177^LuPSMA-I&T, resulting in a PSA-PFS of 12.1 and an OS of 19.2 months. 

In our study, patients showed a PFS of 27 months and an OS of 45 months with a total median of five (IQR 3–6) cycles. The more favorable survival outcome could be partly ascribed to our patient group consisting solely of patients with oligometastatic disease and good performance status [27,28]. The impact of performance status on survival has been previously observed in the context of ^177^Lu-PSMA-RLT. Patients with an ECOG 1 performance status showed a significantly longer OS compared to ECOG 2 (9.7 [7.5–11.9] vs. 6.3 [9.8–12.6]; *p* = 0.0002) [28]. In another study, patients with oligofocal bone metastases showed a significantly longer OS as compared to patients with ≥20 metastases (18.3 vs. 10.8 months; *p* < 0.0001) after standard ^177^Lu-PSMA-RLT [29]. Additionally, the absence of visceral metastasis in our patient group may have influenced the outcome of ^177^Lu-PSMA-RLT in our patient group as depicted by the recently published nomogram [30].

Long survival in oligometastatic mCRPC patients leads to significant absorbed renal doses from the first cycle onward, as the tumor-sink effect is less pronounced [10,11,12,13]. Renal absorbed doses of 0.53 ± 0.21 Gy/GBq were slightly higher than in the dosimetry sub-study of VISION (0.43 ± 0.16 Gy/GBq) [31]. Previous dosimetry-guided studies for ^177^Lu-based Peptide Receptor Radionuclide Therapy explored a safe biologically effective cumulative renal dose of 28–40 Gy [32,33,34]. In our patient group, none of the patients exceeded the safety limit and no severe grade 3 nephrotoxicity could be observed after a follow-up period of 45 ± 21 months [35]. Additionally, hematological grade ≥ 3 adverse events were not observed, which could be attributed to the absence of disseminated/diffuse bone metastasis in our patient group [36]. Moreover, the absence of grade 3 xerostomia in our patient group is in line with large prospective trials, observing only mild to moderate salivary gland impairment in some patients [5].

The retrospective design and small patient group limit the ability to generalize from our results and to perform in-depth statistical analysis. The excellent outcome in patients with a good prognosis, however, permits the application of an intermittent treatment scheme and warrants further, ideally prospective, analysis of this treatment concept. Serious consideration of intermittent ^177^Lu-PSMA-RLT in early responders with oligofocal disease after standard treatment is justified as the late progression to subsequent cycles and dose distribution over long periods can be achieved.

## 5. Conclusions

Intermittent ^177^Lu-PSMA-617 radioligand therapy in early responders with oligometastatic castration-resistant prostate cancer seems to not compromise survival outcome, despite long treatment-free intervals. The safety profile remained favorable, warranting further investigation of the concept of intermittent treatment in selected patients with a longer life-expectancy.

## Figures and Tables

**Table 1:** Patient characteristics at baseline before initiation of ^177^Lu-PSMA-RLT.

	All Patients (*n* = 19)
Age	71 (66–79)
Gleason score:	
<8	5 (26)
≥8	9 (47)
Unknown	5 (26)
ECOG:	
1	18 (95)
2 *	1 (5)
PSA at ^177^Lu-PSMA-RLT initiation (ng/mL)	12.9 (6.4–101.0)
Alkaline phosphatase (U/L)	68.0 (55.0–81.0)
Lactate dehydrogenase (U/L)	214.0 (198.0–272.0)
Disease involvement:	
Only bone metastases	8 (42)
Only lymph node metastases	5 (26)
Lymph node + bone metastasis	6 (32)
Previous treatment:	
Abiraterone	10 (53)
Enzalutamide	9 (47)
^223^Radium dichloride	9 (47)
Chemotherapy with docetaxel	3 (16)

Data presented as median with interquartile range (IQR) or *n* (%); ^177^Lu-PSMA-RLT: ^177^Lutetium-PSMA-617 radioligand therapy; ECOG: Eastern Cooperative Oncology Group Performance Status; PSA: prostate-specific antigen; * without significant comorbidities but a history of deep vein thrombosis and cataract resulting in restricted mobility.

**Table 2:** Hematologic, renal, and salivary gland toxicity grades based on CTCAE v5.0 at baseline, during ^177^Lu-PSMA-RLT (PSMA-RLT), and at follow-up.

Toxicity (Grade)	Baseline (%)		PSMA-RLT (%)		Follow-Up (%)	
	1/2	3/4	1/2	3/4	1/2	3/4
Anemia	14 (74)	0 (0)	16 (84)	0 (0)	13 (68)	0 (0)
Leukopenia	1 (5)	0 (0)	3 (16)	0 (0)	1 (5)	0 (0)
Thrombocytopenia	3 (16)	0 (0)	6 (32)	0 (0)	3 (15)	0 (0)
eGFR *	1 (5)	0 (0)	3 (16)	0 (0)	1 (5)	0 (0)
Xerostomia	0 (0)	0 (0)	1 (5)	0 (0)	1 (5)	0 (0)

* Estimated glomerular filtration rate.

## Data Availability

The datasets analyzed and/or analyzed during the current study are available from the corresponding author on reasonable request.
